# Massive bleeding from a duodenal ulcer in a child with influenza infection: A case report of endoscopic findings

**DOI:** 10.1002/deo2.155

**Published:** 2022-07-17

**Authors:** Kenta Ishimoto, Koichiro Yoshimaru, Yasuyuki Uchida, Keisuke Kajihara, Satoshi Obata, Toshiharu Matsuura, Tatsuro Tajiri

**Affiliations:** ^1^ Department of Pediatric Surgery, Reproductive and Developmental Medicine, Faculty of Medical Sciences Kyushu University Fukuoka Japan; ^2^ Department of Pediatric Surgery Nakatsu Municipal Hospital Oita Japan

**Keywords:** children, duodenal ulcer, hemorrhaging, influenza, laninamivir octanoate

## Abstract

Gastrointestinal bleeding or perforation following influenza infection is rare. We encountered a pediatric case of hemorrhagic duodenal ulcer following influenza A infection. The patient was a 1‐year and 4‐month‐old boy who was diagnosed with influenza A infection and treated with laninamivir octanoate. After inhalation, he had diarrhea, poor appetite, and melena. The next day, he had hematochezia and developed hemorrhagic shock. Contrast‐enhanced computed tomography showed extravasation in the descending part of the duodenum. Esophagogastroduodenoscopy revealed spurting bleeding from a Dieulafoy's lesion on the oral side of the major papilla, and he underwent hemostasis by clipping. From the bulb to the descending part of the duodenum, the mucosa appeared atrophic with spotty redness on the circular folds and multiple and irregularly shaped erosions. Almost all mucosal lesions had healed by the eighth day, and he was monitored as an outpatient for more than one year without re‐bleeding. Intestinal ischemia, viral invasion, and drug reaction of laninamivir octanoate may be involved in duodenal mucosal injury. Acute duodenal ulcers may occur in children with influenza infection, especially young children.

## INTRODUCTION

Although influenza virus infection is generally self‐limited and uncomplicated in healthy children, it is often associated with severe morbidity and mortality. The most common complications of influenza are pneumonia and bacterial infections following sepsis. However, gastrointestinal bleeding or perforation following influenza infection is rare, and only a few severe cases have been reported.

We herein report a pediatric case of hemorrhagic duodenal ulcer following influenza A infection in which hemostasis was successfully achieved with endoscopic treatment.

## CASE REPORT

A 1‐year and 4‐month‐old boy presented with a 1‐day history of fever. He had no significant medical history, allergies, or history of taking non‐steroidal anti‐inflammatory drugs. He was not vaccinated against influenza. He was diagnosed with influenza A infection using a rapid diagnosis antigen test and treated with laninamivir octanoate. After age‐appropriate inhalation of the medicine, he suffered diarrhea and a poor appetite. That night, he presented melena. The following day, he presented with vomiting followed by hematochezia.

**FIGURE 1:**
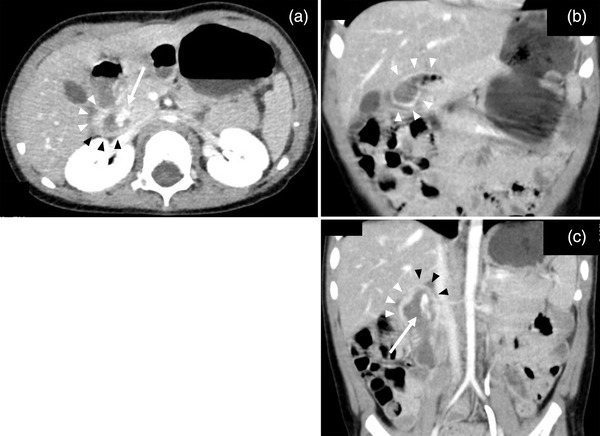
Abdominal contrast‐enhanced computed tomography showing extravasation in the descending part of the duodenum, and the mildly thickened and unevenly enhanced wall of the bulb to the descending part. (a) Axial view. (b, c) Coronal view. Arrows indicate contrast extravasation. Black arrowheads indicate the thickened and weakly enhanced wall and white arrowheads indicate the thickened and strongly enhanced wall

At a general hospital, he showed anemia. Abdominal contrast‐enhanced computed tomography revealed extravasation in the descending part of the duodenum as well as the mildly thickened and unevenly enhanced wall of the bulb to the descending part (Figure 1). During the examination, his blood pressure dropped, and his hemoglobin value decreased from 9.1 to 6.4 g/dL. He was therefore transferred to our hospital with blood transfusion.

On arrival, he was disoriented. His systolic blood pressure was 70–80 mmHg, and his heart rate was approximately 160 beats/min. A physical examination revealed conjunctival pallor and a capillary refill time of 4 s, but no remarkable abdominal findings or skin lesions were noted. Laboratory examinations revealed the following: white blood cell count, 2950/μl; hemoglobin, 12.0 g/dl; platelet count, 118,000/μl; blood urea nitrogen, 29 mg/dl; creatinine, 0.21 mg/dl; aspartate aminotransferase, 75 U/L; alanine aminotransferase, 34 U/L; sodium, 131 mmol/L; C‐reactive protein, 0.11 mg/dl; gastrin, 203 pg/ml; and serum antibodies against *Helicobacter pylori*, < 3 U/ml. Quick stool antigen tests for adenovirus, norovirus, rotavirus, and *Helicobacter pylori* were negative (detail provided in Table S1).

**FIGURE 2:**
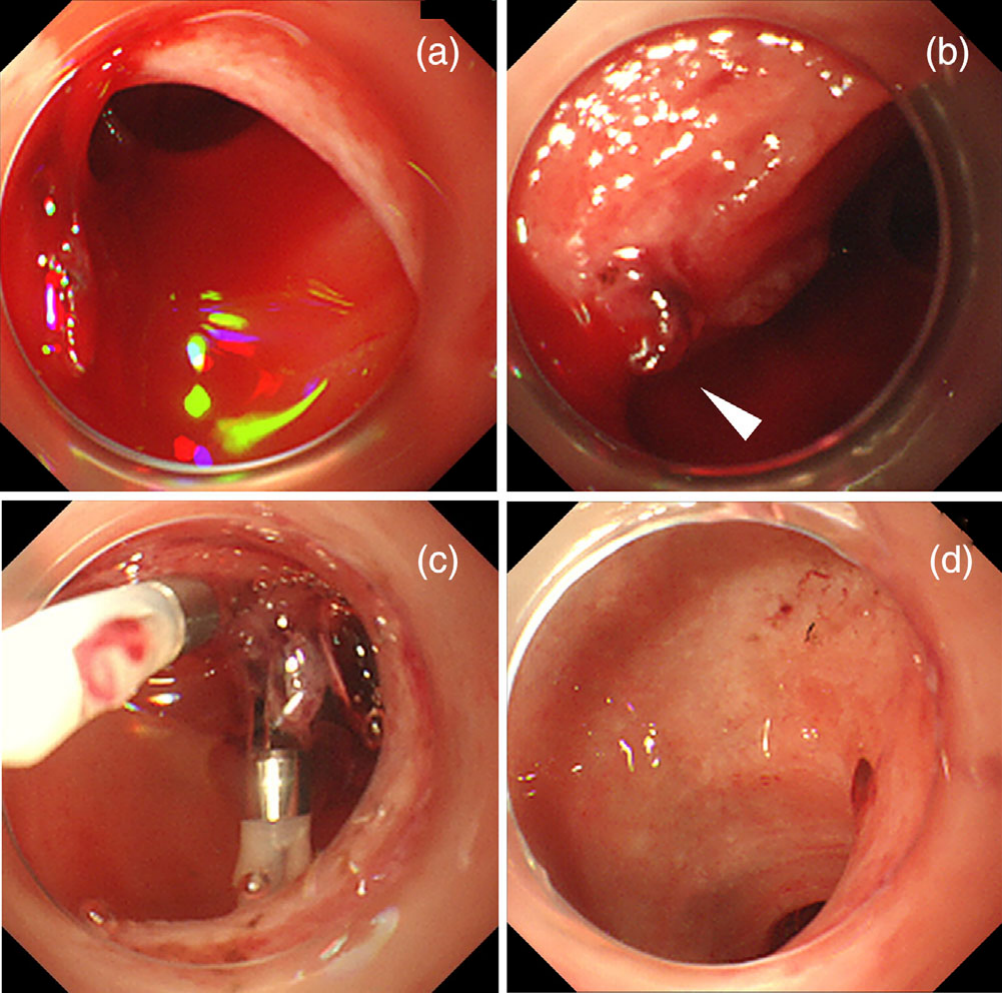
(a, b) Esophagogastroduodenoscopy showing spurting bleeding from an exposed blood vessel (arrowhead) on the oral side of the major papilla. (c) Hemostasis was obtained by clipping. (d) From the bulb to the descending part of the duodenum, the mucosa appeared to be atrophic, and there was spotty redness on the circular folds and multiple irregularly shaped erosions

**FIGURE 3:**
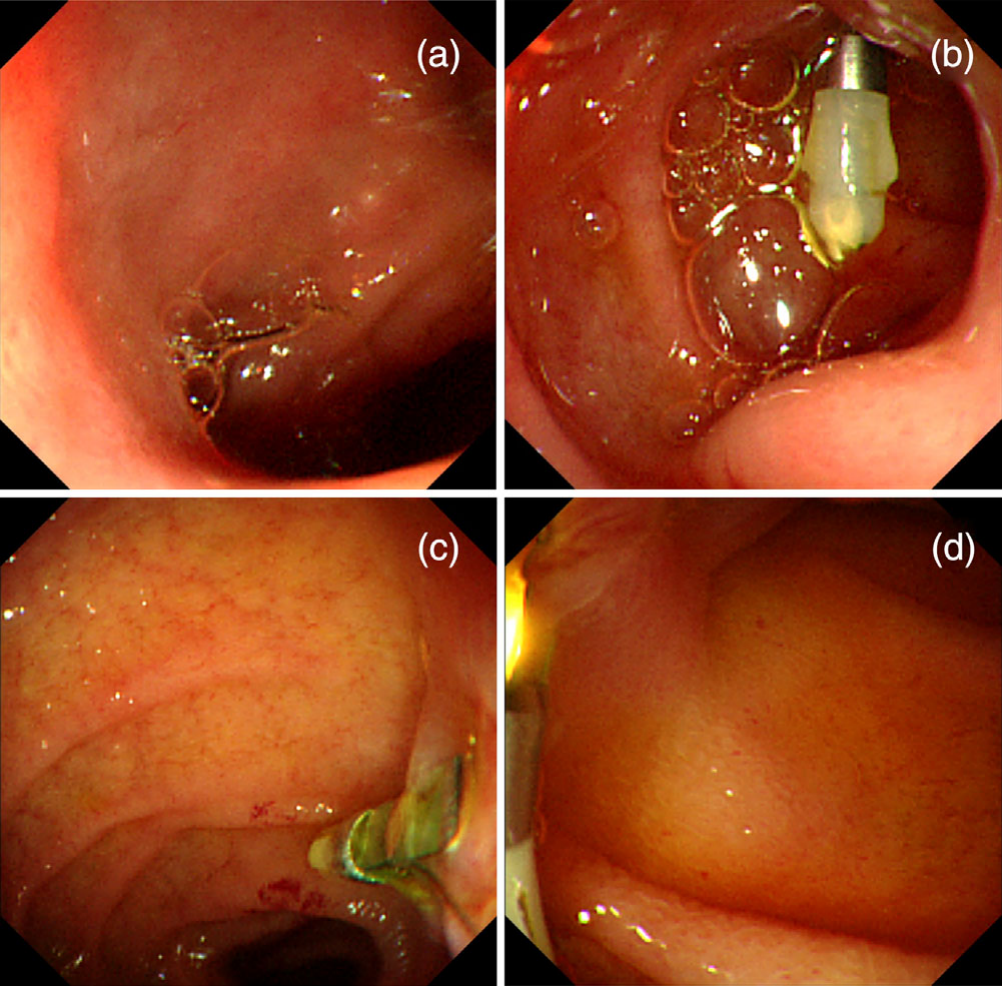
Esophagogastroduodenoscopy on day 8 showed that the duodenal mucosa had healed, with no erosion or redness remaining, except for that caused by contact, and granulation had formed at the clipping site. (a) The bulb. (b) From the superior duodenal angle to the clipping site. (c, d) The descending part, including the clipping site

After his circulation stabilized, emergency esophagogastroduodenoscopy was performed under general anesthesia in the operation room. When the endoscope (GIF‐XQ260; Olympus Co., Tokyo, Japan) with a disposable distal attachment was advanced to the duodenum, we found spurting bleeding from an exposed blood vessel on the oral side of the major papilla, which was considered to be a Dieulafoy's lesion (Figure 2a,b). We performed hemostasis by clipping with two short clips with a claw angle of 90° (HX‐610‐090S; Olympus Co.; Figure 2c). From the bulb to the descending part, the mucosa appeared to be atrophic, and there was spotty redness on the circular folds and multiple and irregularly shaped erosions (Figure 2d). A biopsy was not performed because of the re‐bleeding risk. There were no remarkable findings in the esophagus or stomach other than a shallow mucosal break at the esophagogastric junction. After the administration of a proton pump inhibitor without a combination of mucosal protectant, esophagogastroduodenoscopy on the eighth day revealed that the mucosa had healed, with no redness or erosions noted (Figure 3).

The patient was switched to a histamine‐2 receptor antagonist on day 10, which was continued for one month. We retested his serum gastrin value two months later and found it was within the normal range (106 pg/ml). He has been followed as an outpatient for more than one year without recurrence.

## DISCUSSION

Gastrointestinal hemorrhaging or perforation following influenza infection is rare in children. Although there have been a certain number of reports of peptic ulcers associated with a viral infection, such as norovirus and rotavirus, only 10 cases of peptic ulcers following influenza infection where the lesion could be identified have been reported in the relevant English literature (including abstract only). Regarding duodenal lesions, there have only been five reported cases^1^, ^2^, ^3^, ^4^ (four hemorrhaging cases and one perforation case; Table 1). All cases with hemorrhage involved young children (1–4 years old), and their lesions were in the bulb, while the patient with perforation was 14 years old (lesion details unknown). Notably, in most cases, patients with duodenal lesions developed gastrointestinal symptoms several days after the administration of oseltamivir. Some authors suggested a possible drug reaction, but the association between duodenal ulcers and the drug alone remains unclear.^2^, ^4^

**TABLE 1:** Previously reported cases of duodenal bleeding or perforation in children with influenza infection

									Gastrointestinal symptoms	
No.	Year	Author	Age/sex	Diagnosis	Locations	Treatments	Type of virus	Drugs	Onset	Symptoms
1	2012	Watanabe E et al.^1^	2/M	Hemorrhaging	Bulb	Endoscopy →transcatheter arterial embolization	Unknown	Unknown	Unknown	Melena and abdominal pain
2	2013	Hsueh CW et al.^2^	14/F	Perforation	Unknown	Laparotomy	A	Oseltamivir	Fourth day after administration of the drug	Abdominal pain and vomiting
3	2016	Park CW et al.^3^	4/F	Hemorrhaging (Henoch‐Schönlein purpura)	Bulb	Steroid therapy	Unknown	Oseltamivir	Details unknown after administration of the drug	Abdominal pain and vomiting
4	2018	Masui M et al.^4^	1/M	Hemorrhaging	Bulb (posterior wall)	Endoscopic clipping	A	Oseltamivir	Second day after administration of the drug	Vomiting
5	2018	Masui M et al.^4^	4/M	Hemorrhaging	Bulb (posterior wall)	Endoscopy → laparotomy	A	Oseltamivir	Same day after administration of the drug	Abdominal pain and vomiting
6	2022	Our case	1/M	Hemorrhaging	Bulb and descending part	Endoscopic clipping	A	Laninamivir octanoate	Same day after administration of the drug	Diarrhea, poor appetite and vomiting

Peptic ulcer disease is classified as either primary (intrinsic) or secondary (extrinsic) depending on the etiology, and the etiology and location in children vary depending on age.^5^ Ulcer disease in children under 10 years of age is usually secondary, caused by medication or stress (e.g., respiratory or cardiac distress, sepsis, or dehydration), and is predominantly located in the duodenum. Hsu et al. reported endoscopic findings of children with acute duodenal ulcers, not including those with recurrent or chronic ulceration^6^; 84% of the patients were under 6 years of age and 94% had a preceding illness characterized by diarrhea, upper respiratory infection, or fever. Most of their duodenal ulcers were classified as secondary. On endoscopic examination, more than half of the cases showed multiple shallow ulcers of a heterogeneous size that were distributed over the whole bulb with or without extension into the descending part. These ulcers generally healed rapidly and did not relapse, and were similar to the present case. Considering the endoscopic findings, multiple duodenal erosions were also seen in Henoch‐Schönlein purpura,^3^ which is a small vasculitis in which the vessels of small intestinal villi can be damaged, resulting in necrosis of the villus tip.

With regard to the cause of duodenal mucosal injury in the present case, intestinal ischemia associated with dehydration may be involved. The blood supply is important for mucosal defense mechanisms, and the mucosal blood flow is low in the bulb of the duodenum, suggesting that the duodenum, in particular the bulb, may be more susceptible to ischemia.^7^ Furthermore, damage to the intestinal mucosa from the influenza virus may be involved. Aleandri et al. reported that the influenza virus was able to efficiently replicate in human primary intestinal cells and induce pro‐inflammatory cytokine production.^8^

Recently, several cases of oseltamivir‐associated hemorrhagic enteritis/ischemic enterocolitis have been reported, and the simultaneous occurrence of the following two factors has been considered responsible for the condition: (1) local angiospasm and vasculitis due to the effect of an allergic reaction to oseltamivir; and (2) hypoperfusion in the intestinal tract due to the effect of dehydration caused by influenza.^9^ Suzuki et al. reported a case of laninamivir‐induced ischemic enterocolitis and considered that laninamivir demonstrated the same mechanism as oseltamivir.^9^ Although no detailed information on laninamivir in humans has been reported, approximately 7%–21% is deposited in the lower respiratory tract, and the rest is deposited in the oropharynx after inhaling dry powder zanamivir, of which laninamivir is the C7‐methoxy analog.^10^ It flows into the gastrointestinal tract with swallowing, and its oral bioavailability is poor. The present patient developed gastrointestinal symptoms shortly after inhalation. Taken together, we considered the possibility that this was an allergic reaction.

In the present case, the bleeding point was identified, and endoscopic clipping was successfully performed. Due to the anatomical difficulties of the duodenum in children, it may be difficult to identify the bleeding point and perform hemostasis endoscopically. Interventional radiology or laparotomy should be considered as the next strategy.

The present study was associated with several limitations. We were unable to perform a biopsy and examinations for cytomegalovirus or herpes simplex virus, which can cause duodenal ulcers. We could not sufficiently investigate the cause of duodenal mucosal injury, and we were only able to report that intestinal ischemia, viral invasion, and drug reaction of laninamivir octanoate were possible etiologies. Further accumulation and examination of cases are needed. In conclusion, acute duodenal ulcers may occur in children with influenza infection, especially at young ages, and sudden and severe duodenal bleeding may also occur.

## CONFLICT OF INTEREST

The authors declare no conflict of interest.

## FUNDING INFORMATION

None.

## Supporting information


**Table S1** Laboratory findings on admission
