# Supplementary material for: Massive bleeding from a duodenal ulcer in a child with influenza infection: A case report of endoscopic findings
Source: DEN Open. 2022 Jul 17;3(1):e155. doi: 10.1002/deo2.155 (PMC9307743; doi:10.1002/deo2.155)
Supplement: Supplementary file 1 — Table S1 Laboratory findings on admission [file DEO2-3-e155-s001.docx]

**Table S1. Laboratory findings on admission**

| **Complete Blood Test** | |  |  | **Biochemical Test** | |  |  |  |  |  |
| --- | --- | --- | --- | --- | --- | --- | --- | --- | --- | --- |
| WBC | 2,950 | /μL |  | AST | 75 | U/L |  | IgM | 47 | mg/dL |
| Neutrophils | 43.6 | % |  | ALT | 34 | U/L |  | Antibodies of *H. pylori* | <3 | U/mL |
| Lymphocytes | 47.1 | % |  | LDH | 368 | U/L |  | Gastrin | 203 | pg/mL |
| Monocytes | 5.7 | % |  | T-bil | 0.4 | mg/dL |  |  |  |  |
| RBC | 5.27 | ×10^6^/μL |  | TP | 3.6 | g/dL |  | **Arterial Gas Test** |  |  |
| Hb | 12.0 | g/dL |  | Alb | 2.2 | g/dL |  | pH | 7.36 |  |
| Hct | 34.3 | % |  | BUN | 29 | mg/dL |  | pO_2_ | 70.1 | mmHg |
| MCV | 65.1 | fL |  | Cre | 0.21 | mg/dL |  | pCO_2_ | 29.3 | mmHg |
| MCHC | 35.1 | g/dL |  | Glu | 105 | mg/dL |  | HCO_3_^-^ | 18.1 | mmol/L |
| PLT | 118,000 | /μL |  | CK | 81 | U/L |  | Base excess | -8.3 | mmol/L |
|  |  |  |  | Na | 131 | mmol/L |  |  |  |  |
| **Hemostasis Test** | |  |  | K | 5.0 | mmol/L |  | **Quick Stool Antigen Test** | |  |
| PT% | 62 | % |  | Cl | 106 | mmol/L |  | *Adenovirus* | Negative |  |
| PT-INR | 1.3 |  |  | CRP | 0.11 | mg/dL |  | *Norovirus* | Negative |  |
| APTT | 48.4 | sec |  | Ammonia | 42 | μg/dL |  | *Rotavirus* | Negative |  |
| Fib | 103 | mg/dL |  | IgG | 484 | mg/dL |  | *H. pylori* | Negative |  |
| FDP | 2.5 | μg/mL |  | IgA | 90 | mg/dL |  |  |  |  |

WBC, white blood cell; RBC, red blood cell; Hb, hemoglobin; Hct, hematocrit; MCV, mean corpuscular volume; MCHC, mean corpuscular hemoglobin concentration; Plt, platelet; PT, prothrombin time; PT-INR, prothrombin time-international normalized ratio; APTT, activated partial thromboplastin time; Fib, fibrinogen; FDP, fibrinogen degradation products; AST, aspartate aminotransferase; ALT, alanine aminotransferase; LDH, lactate dehydrogenase; T-Bil, total bilirubin; TP, total protein; Alb, albumin; BUN, blood urea nitrogen; Cre, creatinine; Glu, glucose; CK, creatine kinase; Ca, calcium; Na, sodium; K, potassium; Cl, chloride; CRP, C-reactive protein; IgG, immunoglobulin G; IgA, immunoglobulin A; IgM, immunoglobulin M; *H. pylori*, *Helicobacter pylori*; pH, power of hydrogen; pO_2_, partial pressure of oxygen; pCO_2_, partial pressure of carbon dioxide; HCO_3_^-^, bicarbonate.
